# Acute Stress Alters Auditory Selective Attention in Humans Independent of HPA: A Study of Evoked Potentials

**DOI:** 10.1371/journal.pone.0018009

**Published:** 2011-04-05

**Authors:** Ludger Elling, Christian Steinberg, Ann-Kathrin Bröckelmann, Christan Dobel, Jens Bölte, Markus Junghofer

**Affiliations:** 1 Institute for Biomagnetism and Biosignalanalysis, University Hospital Munster, Munster, Germany; 2 Institute for Psychology, University of Munster, Munster, Germany; Institut National de la Santé et de la Recherche Médicale, France

## Abstract

**Background:**

Acute stress is a stereotypical, but multimodal response to a present or imminent challenge overcharging an organism. Among the different branches of this multimodal response, the consequences of glucocorticoid secretion have been extensively investigated, mostly in connection with long-term memory (LTM). However, stress responses comprise other endocrine signaling and altered neuronal activity wholly independent of pituitary regulation. To date, knowledge of the impact of such “paracorticoidal” stress responses on higher cognitive functions is scarce.

We investigated the impact of an ecological stressor on the ability to direct selective attention using event-related potentials in humans. Based on research in rodents, we assumed that a stress-induced imbalance of catecholaminergic transmission would impair this ability.

**Methodology/Principal Findings:**

The stressor consisted of a single cold pressor test. Auditory negative difference (Nd) and mismatch negativity (MMN) were recorded in a tonal dichotic listening task. A time series of such tasks confirmed an increased distractibility occuring 4–7 minutes after onset of the stressor as reflected by an attenuated Nd. Salivary cortisol began to rise 8–11 minutes after onset when no further modulations in the event-related potentials (ERP) occurred, thus precluding a causal relationship.

This effect may be attributed to a stress-induced activation of mesofrontal dopaminergic projections. It may also be attributed to an activation of noradrenergic projections. Known characteristics of the modulation of ERP by different stress-related ligands were used for further disambiguation of causality. The conjuncture of an attenuated Nd and an increased MMN might be interpreted as indicating a dopaminergic influence. The selective effect on the late portion of the Nd provides another tentative clue for this.

**Conclusions/Significance:**

Prior studies have deliberately tracked the adrenocortical influence on cognition, as it has proven most influential with respect to LTM. However, current cortisol-optimized study designs would have failed to detect the present findings regarding attention.

## Introduction

The impact of acute stress on cognition and sensory processing has been investigated to a lesser extent compared to the consequences of chronic stress. The distinction between the two is, however, important. In some respects, the only aspect shared between both states is the term stress [1]–[3]. Even within the acute stress response, the temporal dynamics of its various aspects should not be neglected [4]–[6]. A coarse subdivision of these aspects may be based on their relative temporal inertness and divided into a first (fast) wave and a second (slow) wave of reactions involved in the entire acute stress response [7].

The term “second wave” mainly refers to altered levels of gonadal and adrenocortical steroid hormones. Among these, the rising secretion of the glucocorticoids cortisol and corticosterone is salient to the extent of providing definitions for the medical concept of stress. The signaling pathway of substances specifically involved in glucocorticoid regulation is commonly referred to as the hypothalamic–pituitary–adrenal axis (HPA). The impact of these hormones on brain function may not occur faster than their rise time. The onset of this rise exceeds several minutes at a minimum. Depending on their diverse mechanisms of cellular action, their effects can outlast their decay, which can take more than an hour. The first wave comprises up- or downregulation of a number of signaling substances that, in turn, regulate the secretion of the aforementioned steroids. Many of these substances cross the blood-brain barrier and exert direct actions on cerebral functions by themselves, bypassing the additional impact of subsequent steroids. These peptides and hormones not only have a relatively fast rise time and half-life, but their actions tend to be rather ionotropic and thus instantaneous as opposed to ligands of the second wave, which predominantly have delayed transcriptional effects [4]. The first wave further comprises activation of the neuronal sympathetic-adrenal-medullary system and the associated secretion of peripheral adrenaline and noradrenaline. Although it does not cross the blood-brain barrier, adrenaline is still suspected to mediate cerebral actions [8], [9]. Independent of these first and second waves of blood-borne neuroactive ligands, acute stress induces specific patterns of neuronal activation within the brain as a third branch of reactions. We will term these patterns intracerebral stress responses and discuss them in more detail below.

In summary, a complete stress response comprises a heterogeneous orchestra of processes, most being capable of influencing cognitive functions. Nevertheless, by far, the majority of research efforts have been devoted to the cerebral backpropagation of the HPA, in particular of glucocorticoids. This mainly pertains to cortisol and, to a lesser extent, adrenocorticotropic hormone (ACTH) as a direct cortisol secretagogue. By comparison, other “paracorticoidal” processes have been fairly neglected. Little is known about the influence of these factors on higher cognitive functions.

Despite the above oblivion, a convincing body of evidence has demonstrated that acute stress elicits an excess of transmission in catecholaminergic systems. Regarding our distinction of fast, slow and intracerebral responses, this falls into the last category. This excess occurs mainly in dopaminergic projections from the ventral tegmental area into prefrontal and anterior cingulate cortices. As opposed to this mesofrontal dopaminergic turnover (MDT), dopamine turnover in nigrostriatal pathways is much less affected. The same holds for other catecholaminergic systems [10]–[16]. Although prefrontal glutamatergic turnover is also increased after acute stress [17], this effect seems to be a secondary consequence of glucocorticoid action [18]. Interestingly, [13] supposed a narrow functionally optimal range of dopaminergic transmission in the prefrontal target regions of MDT. Analogous to a Yerkes-Dodson inverted U-shaped function [19], leaving the optimal MDT range might compromise performance. Taking this together, it is tempting to assume that higher cognitive functions depending on prefrontal integrity, such as working memory, executive functions or attention allocation, are sensitive to disturbance by acute stress. Besides the MDT, similar considerations hold for ascending noradrenergic afferences from the pontine locus coeruleus (LC-NE). Here again, stress-related activation is supposed to affect higher cognitive functions.

The present article focuses particularly on the impact of stress-related catecholaminergic imbalance on selective attention in humans via systems such as MDT and LC-NE. We will extend upon the former as a working hypothesis and address the latter in the Discussion. In a broader scope, this article also aims at promoting scientific interest in causal relationships besides the HPA, as we can exclude HPA to explain our findings.

Stress-related MDT imbalance has already been suspected to impair human selective attention before, as for instance suggested in a review by Arnsten [20]. Currently, however, no data have been used to test the validity of this assumption (however, see the same author's contribution on nonhuman primates [13]). Conversely, an alternative review claimed that stress enhances the attentional focus [2]. The assumption in question comprises two interrelated but separate proposals both awaiting confirmation: first, that acute stress alters the MDT in humans and second the deduction that this alteration impairs the ability to direct selective attention.

The primary proposal regarding the immediate impact of stress on prefrontal dopamine efflux has predominantly been demonstrated using invasive techniques such as microdialysis or intracranial recordings in rodents [12], [14], [21], [17], [22], [23]. This evidence is now undisputed. The validity of rodent animal models for the human prefrontal cortex (PFC) is limited, however, and there have been no subsequent investigations in humans. To a larger extent than other brain structures, the PFC exhibits profound phylogenetic changes between these species [24], [25]. Interspecies differences between rodents and primates also pertain to the mesofrontal dopaminergic system itself [26], [27]. A structure homologous to the human dorsolateral prefrontal cortex (DLPFC) is lacking in rodents [28]. In humans, this area is not notably affected by dopaminergic input from the ventral tegmental area, as opposed to the medial prefrontal cortex (MPFC) [29]. It is this DLPFC, however, that is apparently implicated in cognitive functions such as working memory or attention rather than the MPFC (see [30], [31], for a review). Thus, the second of the above proposals remains questionable: provided that a stress-induced MDT imbalance also occurs in humans, does it impair these cognitive functions?

Current studies using according behavioral tests in humans have reported and reviewed inconsistent findings. Concerning the impact of ecological stressors on working memory and/or selective attention, the general picture also includes examples of improved performance or null results (as in [32], [33] ; but see [34]–[36]). All of these contributions used what might be termed a “lagged design”. That is, subjects were first exposed to a stressor. Subsequent recordings were then deliberately postponed by the estimated cortisol rise time in order to catch the peak. The delayed post-stressor offset varied between 10 and 30 minutes. This approach is neither uncommon nor invalid and reflects the widely accepted definition of stress as threat-related HPA activation. Note that, from a theoretical viewpoint, stress-induced MDT or LC-NE reactions are unlikely to outlast such a delay and to account for these observations. A less frequent suggestion is that a synergistic influence of both the fast and inert wave of the stress response is a prerequisite for the effect under study [34], [37], [38]. Other authors have concluded that both the fast and slow waves could account for their findings independently [39]. In two examples of immediate post-exposition testing, acute stress was found to diminish selective attention as reflected by negative priming [40] and latent inhibition paradigms [41]. In a rare comparison of both immediate and lagged testing, working memory capacity was significantly reduced during the presence of a stressor 15 minutes after its onset, but the effect was no more present 15 minutes after its offset [37]. This is particularly noteworthy as it is a first clue for the influences of fast stress reactions independent of the second wave.

Thus far, we have elaborated on the second proposal that stress impairs selective attention. In the case of evidence for this, it is then a reverse conclusion that remains to be confirmed: impaired performance must convincingly be causally related to the MDT imbalance. Plausible alternative causes are addressed in the Discussion section.

In the present study, we pursued three graded goals. First, we aimed to confirm that acute stress impairs selective attention in humans. We primarily investigated the auditory negative difference (Nd) in a tonal dichotic listening paradigm (DL, [42]). This evoked potential underwent extensive functional validation and may be considered as an electrophysiological indicator of selective attention. Compromising the ability to selectively attend to task-relevant stimuli would reduce the Nd area amplitude (see also the Discussion section).

Second, after the application of a single transient stressor, we sampled a close-meshed, equidistant time series of evoked potentials. Given the short-lived nature of central arousal after stressor offset, electrophysiological indicators of selective attention should decay and recover during a brief period relative to the stress induction. In turn, influences of more inert adrenocortical activity would appear to be sustained after a delayed onset.

Third, provided that the above transient time course emerges, the ascription to a particular fast-acting process among several suspects still has to be made. To this end, we will compare the characteristics of our ERP with ERP in studies having used pharmacological challenges (i.e., dopaminergic agonists) and demonstrate morphological similarities in both the MMN component and the Nd component.

Various pharmacological challenges in humans modulate auditory-evoked potentials related to attention. The Nd has been functionally and morphologically subdivided into an early and a late deflection. There is some consensus that, while the early Nd seems to originate in temporal regions, the later phase also involves frontal sources [43], [44]. Low single oral doses of the D_2_-antagonists haloperidol or droperidol markedly reduce the Nd, which is an effect that is largely restricted to its late phase [45], [46], [43], [47]. Haloperidol also reduces glucose metabolism in prefrontal and anterior cingulate cortices (e.g., [48]). Note that these frontal, but not temporal, sources are affected regarding stress-induced MDT. Whereas the modulation of the Nd components could also be demonstrated for pharmacological challenges of adrenocorticotropic hormone analogues (ACTH 4–10 and ACTH 4–9), no such dissociation of the early and late component was observed here [49]–[52]. These ACTH analogues do not have a corticotropic impact [53], and with respect to corticoids, no effects on the Nd were observed even during continued infusion of 16 mg hydrocortisone in the low point of the HPA circadian cycle [54].

Based on the above three-step line of reasoning, we hypothesized acute stress to first, attenuate the Nd; second, to do so only immediately after application of a stressor; and third, to have a preponderance of the effect in the later and less in the early Nd time range. These phenomena may be interpreted as being progressively indicative of an altered MDT.

Besides an evaluation of the Nd, the dichotic listening paradigm also permits a reanalysis of the same data for the MMN. The MMN was increased by the same haloperidol challenge that already proved to reduce the Nd [45]. An attenuated rather than increased MMN due to ACTH 4–10 intranasal application was reported by [49] (but see [52], for a null result). Hydrocortisone infusion also considerably attenuated the MMN amplitude [54]. As reviewed above, in the same study, this did not modulate the Nd. The synopsis of these findings permits a further distinction of influences in our data. Besides a selective decreasing effect on the late Nd, a further clue for MDT would be an increased MMN. A trend in terms of MMN reduction, in turn, would rather point to consequences of HPA-related ligands. This reduction being temporally coincident with a reduced Nd would further allow us to track the critical aspect of the HPA to ACTH rather than to glucocorticoids. Under the premise of MDT as a working hypothesis, a conceivable outcome was an initial MMN amplification that eventually gives way to decline, as the post-stressor central arousal decays and adrenocortical output rises over time.

## Methods

Forty-three adult subjects were recruited via local advertisements. Screening criteria comprised drugs of abuse including nicotine, current medication with hormone preparations, beta-adrenergic antagonists or psychopharmaceuticals and diagnosis of impaired hearing or psychiatric conditions. As counterindications for cold pressor stress induction, cases of epilepsy, cardiovascular diseases, hypertension or diabetes mellitus were also excluded. To avoid influences of the ovarian cycle on adrenocortical reactivity, only men were included (see [55], [56], for review). Subjects were instructed to refrain from caffeine on the day of recording and from ample meals, juice or candy one hour beforehand. Nine subjects were discarded post-hoc due to equivocal statements on substance abuse, self-determined abort during the stressor application, task default or artifactual recordings. In one additional subject, only the recordings of electrodermal activity (EDA) failed. The findings below are based on N = 34, aged MN = 23.8 years (SD = 3.5) or N = 33 for EDA.

### Ethics statement

The subjects gave written informed consent prior to participation and were individually debriefed thereafter. Approval from the ethics committee was granted at the University of Konstanz.

### General time course

**Figure 1:**
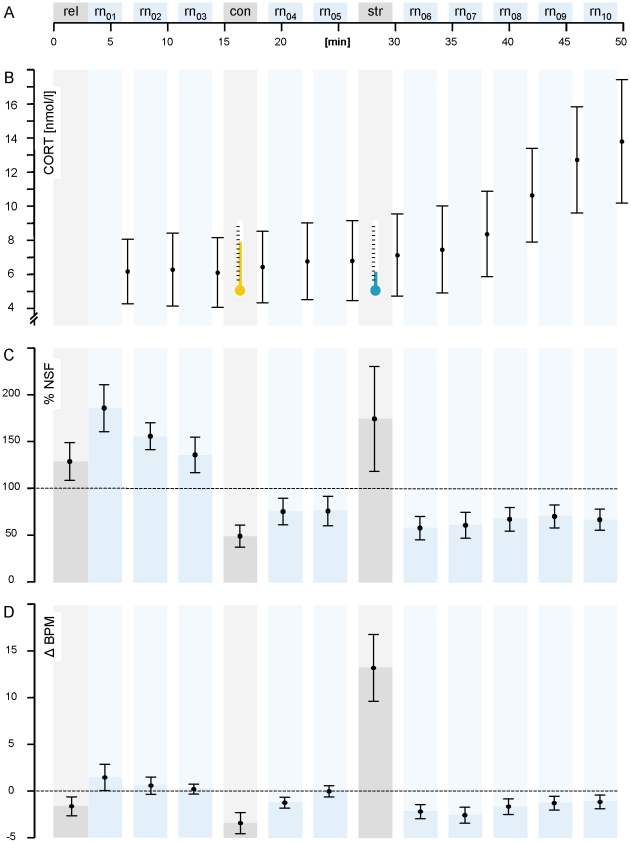
Time course of experimental stages and parameters of stress. Panel A: Timeline of experimental procedures with *rel* = initial relaxation, *con* = control procedure, *str* = stress induction procedure and *rn*_#_ = recording runs with EDA, EKG (as depicted below) and EEG (as depicted in Figure 3). Dichotic listening tasks were performed during these runs. Blanks indicate breaks used for saliva sampling. Panel B: Time course of salivary cortisol concentration in nmol/l. For sAA concentrations, see the Results section. Panel C: Deviance of NSF from individual grand average for single runs in percent. Panel D: Deviance of HR from the individual grand average in BPM. Whiskers delineate confidence intervals of p = .95.

After the receipt of instructions, the completion of a consent form and a health declaration and the technical preparation, subsequent steps were then conducted at a schedule-pace. Timing relative to the stressor was thus aligned for each subject. In an initial relaxation phase, a soothing piano sonata and a video were presented. A series of ten DL-task runs followed, which were interrupted by a control procedure and a stress induction after runs 3 and 5, respectively. Each of these steps (DL-tasks, stress induction, control) lasted for 180 seconds and was followed by a break of 60-seconds. The counterbalancing of condition orders was waived in order to prevent crosstalk from endocrine reactions in the control condition (cf. [57]). Instead, three introductory runs permitted control for habituation or sensitization trends and permitted such trends to saturate. Refer to Figure 1A for a timing scheme of the experimental steps.

### Stressor

The stress induction protocol consisted of a single cold pressor task (see [58], for a review of validations). Subjects were aware of potential cold and warm foot baths to come, but did not know the number, order or duration prior to the recordings. During the stress induction, subjects were then prompted to submerge their bare feet up to the ankles in cold water (3°C) and, after 180 seconds, to withdraw them. In the case of apparently unresponsive subjects, the water was additionally stirred. The control procedure was similar with warm water.

### Dichotic listening task

The subjects were dichotically presented with two independent streams of standard tones (800 and 1500 Hz) with interspersed deviants (840 and 1560 Hz) at a ratio of 1∶9 per stream. The tones were at 60 dB (SPL) with a ramp time of 2 times 5 ms and a 40-ms plateau. The common inter stimulus-interval (ISI) for both streams was equally distributed within [250∶1250] ms (thus, a mean ISI of 750 ms). Subjects were instructed to silently count the occurrence of deviants in one stream while ignoring the other side and to report the sum at the end of the run. The performance verified the proper compliance of all subjects involved, sparing further rejections. The behavioral data are provided in Table S1. The frequency (800 Hz/1500 Hz) and the side to attend to (left/right) was permuted over four blocks within each run, and the respective block order was counterbalanced over subjects in a Latin square scheme. Per run, the average number of trials per subject and condition (attended or ignored tones) was 89.5 after artifact rejection. Tones were presented via closed back supra-aural headphones with a frequency range of 0.02 to 16 kHz (PC Headset 120, Logitech, Romanel-sur-Morges, Switzerland). Inversion of the headphones was waived, as lateralization effects were of no particular interest.

### EEG recording

During the DL-task, an EEG was collected with 64 Ag/Ag–Cl electrodes according to the extended international 10/20 system using an integrated amplifier-digitizer system (AMB-TRF72AB and ASA-lab, Advanced Neuro Technology, Enschede, The Netherlands). Additional electrodes were placed at the left and right mastoids, but the analyses were based on the common average reference. The ground electrode was positioned at the midline of the forehead. Impedances were kept below 10 kΩ. Hardware low pass filtering and digitizing were carried out at a 138-Hz cutoff and 512 Hz, respectively. Data preprocessing was conducted using BESA 5.2, Megis GmbH, Gräfeling, Germany. The processing steps were comprised of artifact correction, offline filtering at an 80-Hz low pass (24 dB/oct) and a 0.3-Hz high pass (6 dB/oct) and artifact rejection (gradient>14.6 µV/ms, peak-to-peak amplitude>120 µV/ms). Artifact correction was performed using a two-stage spatial filtering method based on the electrocardiogram (ECG) and vertical oculogram (see [59], for a detailed account). Compromised channels were interpolated if applicable, provided that they were not adjacent and that the overall number was less than four. The average number of channels interpolated was 0.6. Averaging epochs comprised a [−200:0] ms baseline interval used for correction. Averaged data were evaluated using in-house software (EMEGS 2.3, Junghofer and Peyk, 2004) running under MATLAB 7 SP3 (The MathWorks, Natick, MA, USA).

### EEG evaluation

**Figure 2:**
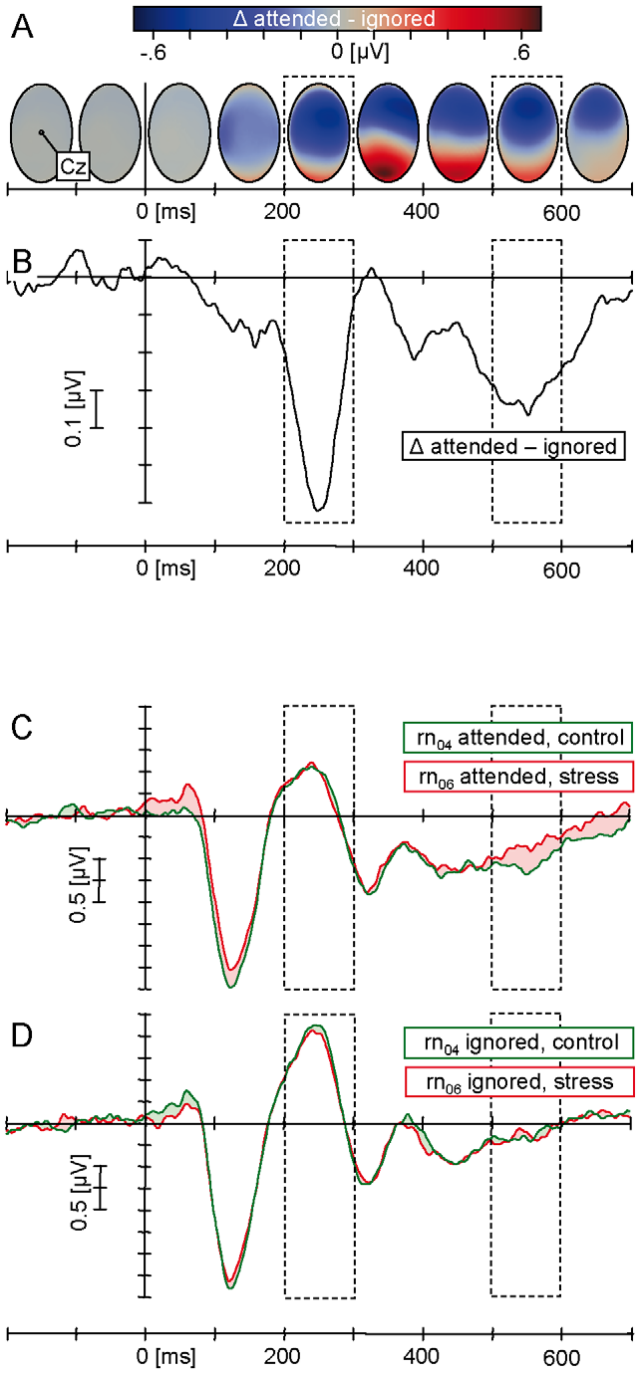
Morphology and topography of the Nd and stress-induced modulations. Upper two panels: Time course of the Nd difference topography and morphology. Data represent the grand average of evoked responses to attended minus unattended tones including all runs. Note the intermediate shift of topographical zero crossing towards the coronal midline in panel A, driving the bimodal appearance of the Nd in panel B, which in turn depicts the average referenced vertex potential (Cz). The ROI (cf. Figure 2) and time bins (dashed boxes) were selected on the basis of these visualizations A and B to be used for area measures in the statistical analysis and the depictions in panels C, D and Figure 3. Lower panels: Comparison of evoked responses to attended (panel C) and ignored (panel D) tones in the runs rn_04_ (subsequent to the control procedure, green lines) and rn_06_ (subsequent to the stress induction, red lines). Signals refer to the ROI as determined by panel A. Negative is plotted downward throughout.

**Figure 3:**
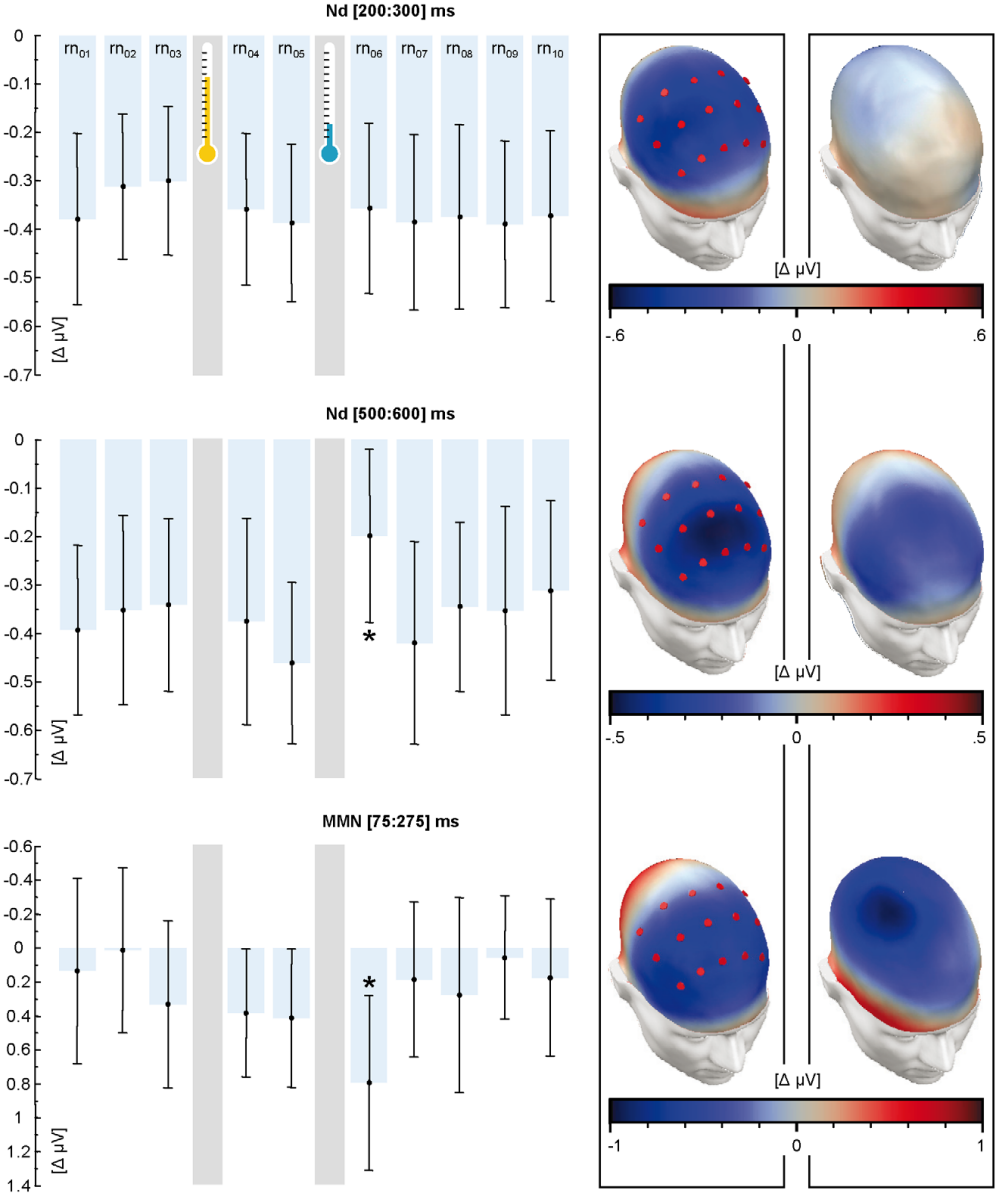
Topography of ERP difference components and time course of amplitudes. The top to bottom panels refer to the early Nd, late Nd and MMN, respectively. The bar charts depict area measures of amplitude (see the Methods section) for subsequent runs. The whiskers delineate p = .95 confidence. The left-hand maps show grand average difference topographies over rn_01:10_; sensors included in the ROI are highlighted in red. The computation of area measures was based on these ROI. The right hand maps show the deviation of these topographies in rn_06_ (subsequent to the cold pressor) from the grand average of the remaining runs.

The Nd was calculated as the mean difference in amplitude between area measures for attended minus unattended tones. Following prototypical morphology reviewed by [44], latency bins were determined as intervals centered around local difference maxima at [200∶300] and [500∶600] ms (Figure 2B). In accordance with previous reports, a restricted set of 15 fronto-central leads constituted the topographical maximum (Figures 2A and 3) and was thus subjected to subsequent analysis (Fz, FCz, Cz, F2, FC2, C2, F4, FC4, C4, and left corresponding; c.f. [44]). The MMN was identified based on guidelines provided by Duncan et al. ([60], see also [44]) as negativity within a [75∶275] ms latency interval of the subtraction signal (deviants minus preceding standards). Among all sensors anterior to the coronal midline [60], a ROI of 15 leads was selected based on the maximum difference topography over this time range (coincident with the Nd sensor set). For the sake of comparability (see preceding reports by [45], [49]), MMN was determined for unattended tones only. This and the restriction of the interval to 275 ms were also aimed at preventing contamination with P2b or P3 components. (See [61], [54]). For both Nd and MMN intervals, ipsative area measures were then used for further visualization and statistics.

The data were then subjected to a supplementary reanalysis in terms of a distributed source model using a least square minimum norm criterium. The methods and the results are presented and discussed in detail in Text S1 and Figure S1.

### EKG and EDA

A bipolar lead ECG was recorded simultaneously to the EEG and evaluated for heart rate (HR). Likewise, EDA was recorded hypothenar on the non-dominant hand using Ag/Ag-Cl electrodes with a 6-mm diameter of the active area (Varioport, Becker Meditech, Karlsruhe, Germany). The nonspecific fluctuation frequency (NSF) and skin conductance level (SCL) were derived. Both physiological measures were preprocessed and evaluated using ANSLAB 2.4 (University of Basel, Institute for Psychology, Switzerland) running under MATLAB 7 SP3, then averaged per run and normalized to individual grand averages.

*Saliva samples*. Samples were collected in-between runs using Salivette® (Sarstedt AG, Numbrecht, Germany) and were then centrifuged at >1000 g for 2 min and stored at <18°C. In a single lot, free cortisol and alpha amylase concentrations (sAA) were determined externally by the Institute of Biopsychology of the Technical University Dresden, Germany. Cortisol was quantified by means of a commercially available enzyme-linked immunosorbent assay (IBL International GmbH, Hamburg, Germany) with a lower detection limit of 0.41 nmol/l. Quantification of sAA was done spectrophotometrically with an enzyme kinetic method (α-amylase EPS Sys; Roche Diagnostics, Mannheim, Germany), irrespective of flow rate. Further technical details are available at [62]. Six pairs of concealed aliquots confirmed the high accuracy of the blind analysis for cortisol with an average intra-assay coefficient of variation of 5.73%. For sAA, this coefficient was 18.01%.

## Results

Recording runs will be referred to by their relative position as *rn_01_*, *rn_02_*, ..., *rn_10_*. The stressor *str* was administered between *rn_05_* and *rn_06_*, and the control procedure *con* was administered between *rn_03_* and *rn_04_*. Saliva samples are referred to by the preceding step, that is, *str* indicates the sample between *str* and *rn_06_*. T-tests are single tailed unless this is stated otherwise.

### Electrodermal activity and heart rate

Both physiological recordings verified the stressor and also revealed a wide variability in responsiveness between subjects. During *str*, on average, there was an individual increase in heart rate of 16.6 beats per minute (BPM, SD = 11.1) relative to *con* with F_(1,33)_ = 74.375, p<.001. That is the HR increased by 24% relative to the control procedure. Convergent with the fact that cardiovascular markers of adrenomedullary and vegetative activation decay rapidly after the cessation of stress exposure [63], differences in HR were not present in the immediately subsequent *rn_04_* and *rn_06_* (Figure 1D). Cognitive loads such as those involved in a DL-task may also increase the HR as compared to resting states [63]. This is reflected by the fact that, during *con*, when subjects were idle, the HR was evidently lower than during the mild task demand during the recording runs (Figure 1D). Although this does not compromise the data, it shows that there is no single correlate of stress providing full discriminant validity and unaffected by contaminating factors [64]. Likewise, cardiac measures may also be affected by confounding thermoregulatory reactions to a cold pressor [65]. It is thus important to not rely on single markers of stress, but here, the SCL was also increased in *str* as compared to *con* (F_(1,33)_ = 10.747, p<.01), as was the NSF (F_(1,33)_ = 17.325, p<.001). Successful stress induction as a prerequisite for the intended principal analysis is thus confirmed, and we have no further questions addressing the physiological data.

### Salivary samples

From *str* to the final *rn_10_*, the individual rise in cortisol concentration was MN = 6.42 nnol/l (SD = 8.17). The timing was in line with the known kinetic profile (Figure 1B and [66]). A linear trend over *str* to *rn_10_* resulted in F_(1,30)_ = 22.014, p<.001. The sAA concentration remained stable throughout the experiment without any noticeable trends. The sAA peak concentration after stress induction occurred at *str* with MN = 85.9 U/ml (SD = 62.4) whereas the grand average of *rn_01:10_* was 83.5, which is a difference that is clearly below the error in the assay (see Methods). Although this timing of the peak concentration meets our expectations, a planned contrast of *str* vs. all other runs *rn_01:03;con;04:12_* was not significant with F_(1,25)_ = 0.015, p = .904. The same holds for a test of the post-stress vs. post-control samples rn_04_ vs. rn_06_ with F_(1,30)_ = 1.355, p = .254.

### Auditory event-related potentials

Concerning the ERP, Figure 3 shows a steep decline of the late Nd and a simultaneous incline of MMN after stress induction in *rn_06_*. Both effects were no longer present in *rn_07_*. An a priori planned contrast of *rn_06_* vs. all other runs *rn_01:05;08:10_* was significant for both the late Nd (F_(1,33)_ = 6.36; p<.05) and MMN (F_(1,33)_ = 5.496, p<.05), but not for the early Nd (F_(1,33)_ = 0.001, p = .982). The interaction between the Nd interval (early vs. late bin) and the preceding treatment (*rn_04_* vs. *rn_06_*) was marginally significant, with F_(1,33)_ = 3.400; p = .074. The direct comparison of the runs following control (rn_04_) and stress induction (rn_06_) for the late time bin was significant with T_(33)_ = 1.92; p = .032. This was not the case for early bin, with T_(33)_ = 0.257; p = .399. A reanalysis of the data in terms of a source space reconstructions is presented in Text S1 and Figure S1. In summary, our prognoses for fast, transient modulations of the Nd and MMN were met.

Over subsequent runs with increasing distance to the stressful event (*rn_07_* to *rn_10_*), no gradual trend, which would have been indicative of HPA-driven modulations, was observed in any component. More specifically, linear contrasts failed significance for the early (F_(1,33)_ = 0.004, p = .948) and late Nd (F_(1,33)_ = 0.425, p = .519) as well as for the MMN (F_(1,33)_ = 0.048, p = .828). Therefore, our prognoses for inert, slowly rising modulations of the Nd and MMN were not met.

There is notable unsystematic variability in the MMN over the runs as compared to the Nd (Figure 3). This is due to the stimulation protocol and, in particular, to the deviant frequency. It was deliberately optimized for the Nd, which came at the expense of the signal-to-noise ratio of the MMN (c.f. [43]). Nevertheless, the effects outweighing such competing variance as above are credible.

## Discussion

Generally, the term selective attention refers to the ability to filter out irrelevant perceptions to the benefit of relevant ones under conditions of competition for restricted processing resources [67]. The reactive spontaneous access of external stimuli to such resources due to their salience is termed exogenous attention. Endogenous attention refers to a volitional, goal-driven selection. Here, we defined the term selective attention more specifically as competitive predominance of endogenous over exogenous attention.

A tonal DL-task as used here creates a competition for limited processing resources among both latent systems. According to its extensive functional validation, the manifest Nd is a correlate of endogenous attention [68], [44]. The MMN reflects processes involved in the initiation of exogenous attention [69], [61]. A reduced relative prevalence of endogenous attention should be reflected in an attenuation of the Nd. Our findings clearly point to this posited and evident increase in distractibility. The time course of a quick rise and decay also matches the prior model-based expectancies.

Another more detailed claim has to be considered more tentatively, however: We interpret the driving latent process behind this as stress-induced activation of dopaminergic projections from the ventral tegmental area into prefrontal and anterior cingulate cortices, which we coined MDT. Indeed, the observed modulations of the evoked potentials exhibit the pattern known from drugs acting as dopaminergic ligands with respect to Nd as well as MMN. Similarly, we can preclude ACTH and corticoids based on their known characteristic ERP modulations. This reasoning, however, depends on the reliability of premises derived from pharmacological studies. Admittedly, our deductions are based on a small body of literature. In particular, the selective functional sensitivity of the late Nd to dopaminergic challenges needs further confirmation.

Furthermore, there are several alternative causal attributions that cannot be discarded at this point. Let us now consider these potential confounding factors. The most important is a substantial change in discharge patterns of noradrenergic projections emanating from the locus coeruleus (LC-NE). This change is another important aspect of the intracerebral stress response [70]–[72]. In fact, the LC is among the most stress-sensitive brain structures [73]. In an exemplary study, Alexander et al. [74] attributed cognitive effects to stress-induced activation of LC-NE by means of a propranolol challenge. [75] also interpreted their findings on emotional attention under acute stress as related to LC-NC activity.

Moreover, stress-related LC-NE activity is regulated by extrahypothalamic corticotropin releasing factor (CRF), which also exerts more direct actions on limbic structures [76]–[78]. As opposed to this extrahypothalamic CRF, neurocrine CRF in the pituitary portal circulation is not directly neuroactive, as it does not pass through the blood-brain barrier [1], [79]. However, other stress-related neurocrine peptides do and also affect cognition [80], [81]. Hence, a number of interpretations of our findings that involve processes other than MDT are viable, provided they have a comparable temporal dynamic. This remains beyond the scope of this study.

More distinctly, the present data rule out the influence of downstream stages of the HPA on the basis of effect latency. This, as the authors themselves point out, is not covered in the otherwise conclusive evidence of Alexander et al. [74]. Although ACTH is secreted quickly after the advent of a stressor, its effects on ERP arise much later. Regarding the temporal dynamics of ACTH 4–10 action with some temporal resolution, [53] found a time lag of 10–30 minutes for the modulation to develop after a single bolus of 1 mg, which may be interpreted in terms of delayed metabotropic or transcriptional signaling pathways. Thus, HPA activation does not account for our volatile effects.

The fact that no effects of ACTH, even in our later recording runs, were observed is somewhat unexpected. We offer the explanation that the latest recordings were terminated 20 minutes after the stressor. The electrophysiological effects of the ACTH 4–10 challenges reviewed above stem from studies using a lagged design of consecutive substance administration and testing. Delays ranged between 30, 40 and 60 minutes after intranasal [49], intravenous [50] and oral administration [51], respectively. However, our explanation remains speculative, as there is a shortage of investigations using high-resolution time series.

Given the absolute increase in cortisol concentrations, it is also possible that the respective ACTH response did not reach some speculative critical limit in our case. The preponderance of sympathico-adrenocortical reactions to a CP has already been discussed by Schwabe et al. [82] (see also [83]). Although the cortisol reaction to our CP is about three times as high as in comparable studies (e.g., [82], [84], see also [83]), it falls short of more potent social evaluative stressors. Here, a common finding could be about twice this amount [85], [86], [66]. Pharmacological studies, as reviewed above, even tend to exceed physiological doses (but see [52]). This might explain why their findings do not agree with out data.

To summarize, the current study's outcomes are threefold. There is sufficient evidence for the supposition that stress impairs selective attention. Furthermore, there is tentative evidence that MDT causes the resulting effects among all of the candidate factors outlined above. Importantly, there is marked evidence that a causal role of HPA is unlikely.

As reviewed above, the long-term effects of HPA activation have attracted major interest in the present research and debate. Given their latency, prior investigations commonly use a time-lagged design of consecutive stressor exposition and data recording. It is evident that the present findings would have escaped such an approach. Besides the immediate question under study, our report also aims at stimulating the discussion with a different methodological scope. For future studies on the impact of stress on cognitive functioning, we offer three suggestions. First, we deem attentive consideration of both HPA and non-HPA causality equally important. Second, the temporal dynamic of stress-related cognitive changes deserves particular interest. These may not only differ in latency for different substances, but they may even be inversed for single substances over time [87]. Third, such non-monotonic response curves also pertain to the topic of dose dependency [88], [87], which we addressed only superficially. Inconsistent findings in current research might be explained in terms of these topics. As the term stress refers to a heterogeneous construct, differentiated investigations seem promising.

## Supporting Information

Figure S1 All depictions show subtractions of attended minus unattended stimulation. Whiskers delineate confidence intervals of p = .95. Panel A: Topography of the difference source activity in a time range of [100∶600] ms. A bilateral temporal and a frontopolar dipole cluster were selected based on this topography as ROI. These are the basis of the below panels and subsequent analyses. Panel B: Global Power of the Nd source activity in the temporal (green) and frontal (blue) ROI. Time bins of [100∶200] ms and [400∶550] ms were selected based on the bimodal maxima of the joint activity of both ROI (dashed boxes). Influences of stress do not differentially affect the early and late Nd. Thus, Panels C and D depict unweighted mean activity over both bins. Panel C: Activity of the frontal Nd generator during consecutive runs. Note the drop of Nd amplitude after stress exposition. By comparison, the activity of the temporal generator (Panel D) remains constant. This pattern occurs without great difference in both latency intervals (Panel E).
(TIF)

Table S1 Itemization of the individual performance in the dichotic listening task. The numbers indicate the deviation between the correct solution and the subject's reply.
(PDF)

Text S1 A reanalysis of the EEG data using a pseudoinverse calculation.
(TIF)
