# Supplementary figures and images for: Acute Stress Alters Auditory Selective Attention in Humans Independent of HPA: A Study of Evoked Potentials
Source: PLoS One. 2011 Apr 5;6(4):e18009. doi: 10.1371/journal.pone.0018009 (PMC3071695; doi:10.1371/journal.pone.0018009)

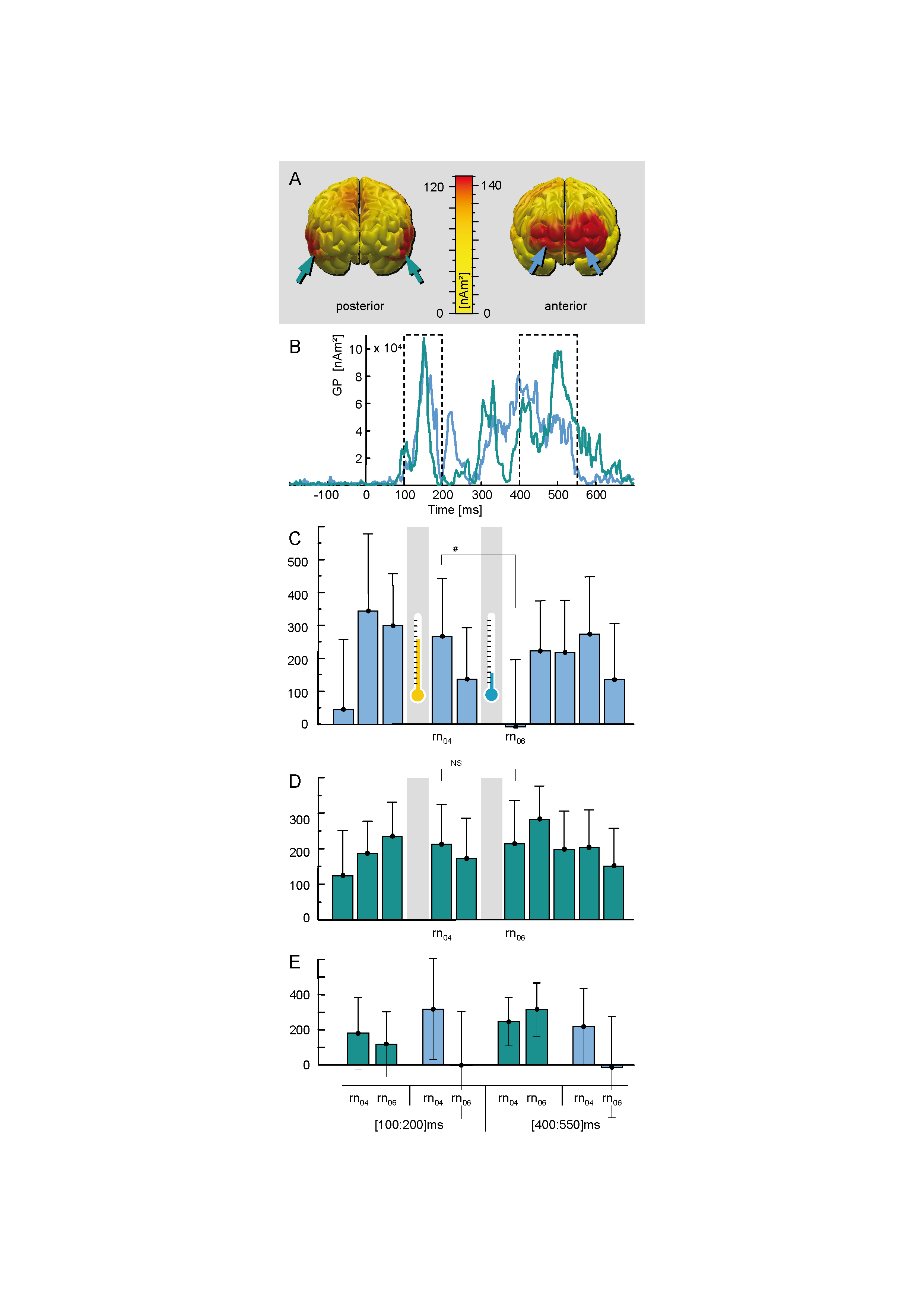

Supplement: Figure S1 — All depictions show subtractions of attended minus unattended stimulation. Whiskers delineate confidence intervals of p = .95. Panel A: Topography of the difference source activity in a time range of [100∶600] ms. A bilateral temporal and a frontopolar dipole cluster were selected based on this topography as ROI. These are the basis of the below panels and subsequent analyses. Panel B: Global Power of the Nd source activity in the temporal (green) and frontal (blue) ROI. Time bins of [100∶200] ms and [400∶550] ms were selected based on the bimodal maxima of the joint activity of both ROI (dashed boxes). Influences of stress do not differentially affect the early and late Nd. Thus, Panels C and D depict unweighted mean activity over both bins. Panel C: Activity of the frontal Nd generator during consecutive runs. Note the drop of Nd amplitude after stress exposition. By comparison, the activity of the temporal generator (Panel D) remains constant. This pattern occurs without great difference in both latency intervals (Panel E). (TIF) [file pone.0018009.s001.tif]
