# Supplementary material for: Acute Stress Alters Auditory Selective Attention in Humans Independent of HPA: A Study of Evoked Potentials
Source: PLoS One. 2011 Apr 5;6(4):e18009. doi: 10.1371/journal.pone.0018009 (PMC3071695; doi:10.1371/journal.pone.0018009)
Supplement: Table S1 — Itemization of the individual performance in the dichotic listening task. The numbers indicate the deviation between the correct solution and the subject's reply. (PDF) [file pone.0018009.s002.pdf]

	run									
subject ID	rn01	rn02	rn03	rn04	rn05	rn06	rn07	rn08	rn09	rn10
01	1	0	0	1	0	3	1	0	0	0
02	0	1	0	0	0	1	1	1	0	0
03	1	0	0	0	0	1	1	0	0	0
04	1	4	1	1	3	2	2	0	1	1
05	2	1	3	0	1	2	0	0	2	1
06	2	2	0	1	1	0	2	1	2	3
07	4	4	0	2	0	3	3	2	5	2
08	1	1	1	3	4	3	1	1	2	2
09	1	4	0	0	0	1	0	0	2	mv
10	3	1	1	1	0	1	1	0	0	1
11	2	2	1	1	0	4	4	0	1	0
12	5	1	0	0	1	2	0	1	2	0
13	3	5	2	5	1	3	2	0	3	3
14	4	5	2	0	0	0	2	0	0	1
15	4	1	2	1	0	4	1	1	0	1
16	6	1	1	0	0	0	0	0	0	0
17	1	3	1	3	3	2	2	1	0	0
18	5	1	0	1	0	0	0	0	0	1
19	1	0	0	1	0	2	0	0	0	1
20	4	1	2	2	1	2	3	0	1	1
21	2	5	1	5	2	1	2	3	1	1
22	1	2	1	1	0	2	2	0	2	0
23	1	1	1	0	0	0	1	0	0	0
24	1	1	0	1	3	1	1	1	1	0
25	5	4	2	4	0	0	1	3	6	1
26	2	1	1	3	3	4	1	2	3	1
27	1	1	1	1	1	1	0	0	2	2
28	3	2	2	4	1	3	2	3	2	4
29	1	4	1	4	0	2	4	2	4	1
30	2	0	1	3	1	3	1	1	3	1
31	0	1	1	1	0	1	1	1	0	1
32	1	3	0	1	0	0	1	1	2	0
33	2	2	2	6	0	3	2	2	3	1
34	0	2	3	4	0	1	5	1	2	3
