# Supplementary material for: Acute Stress Alters Auditory Selective Attention in Humans Independent of HPA: A Study of Evoked Potentials
Source: PLoS One. 2011 Apr 5;6(4):e18009. doi: 10.1371/journal.pone.0018009 (PMC3071695; doi:10.1371/journal.pone.0018009)
Supplement: Text S1 — A reanalysis of the EEG data using a pseudoinverse calculation. (TIF) [file pone.0018009.s003.tif]

A reanalysis of the EEG data using a pseudoinverse calculation.

Methods

The single averages were subjected to a pseudoinverse calculation based on a sample-wise minimum norm least square criterion (e.g., [1]). The brain geometry was approximated by a 160 mm diameter sphere with 350 tangentially rotating superficial dipole vectors fixed in equidistant positions. The principles of this procedure are similar to the more detailed description of [2], except for a fixed regularization parameter of $\lambda = .03$ that was used in our case. Separate inverse calculations were applied to the respective AEP of attended and unattended tones. Similar to the processing of the raw sensor data, further evaluations were then based on difference waveforms of the estimated source signal.

A topographical map of an interval of [100:600] ms was then used for an identification of ROI that are affected by main effects of attention (Figure S1A). Analogous to prior reports (cf., [3,4]) centers of activity were found on both hemispheres in the temporal cortical areas and another center at a frontopolar location. Note that the inverse calculations were based on a spherical head model. The projection on a more realistic brain geometry (as depicted in Figure S1A) is meant to enhance the intelligibility of the figure rather than the spatial precision. A cluster of 6 dipoles was then positioned in the center of the temporal ROI and another equally sized cluster was positioned in the frontal ROI. Source waveforms of both regions were used for the extraction of area measures of signal amplitude. Similar to the procedure that was applied to the raw sensor data, the latency bins were centered around local maxima at [100:200] and [400:550] ms (Figure S1B).

Results

The bimodal course of the Nd source waveform parallels the morphology of the same raw data in the sensor space. However, in the source space, the latency of the peak activity was shorter (cf. Figures 1B and S1B). Over both ROI and time bins, there was a difference between attended minus unattended stimuli ($F_{(1,33)} = 85.824$, $p < .001$). There was no apparent difference between both ROI. An interaction of attention direction times region was not significant ($F_{(1,33)} = 1.252$, $p = .271$). The Nd was stronger in the late as compared to the early time bin, the interaction of the attention direction and time bin resulted in ($F_{(1,33)} = 11.34$, $p < .01$). A transient drop of the ipsative Nd after the stressor exposition, such as the one that was observed in the late sensor-based Nd (Figure 3, middle panel), also occurred in the reconstructed Nd of the frontal generator (Figure S1C).
Stress-related modulations of the Nd did not affect the two time bins differentially (Figure S1E). Moreover, although both bins were equally affected by the stress induced modulation of the Nd, this modulation was wholly restricted to the frontal pole (Figure S1C-E). The difference of the frontal Nd at rn_{04} vs. rn_{06} was marginally significant ($T_{(33)} = 1.327$, $p = 0.096$). In the temporal ROI, there was no such modulation and the same test was not significant ($T_{(33)} = .023$, $p = 0.49$).

Discussion

The source analysis of the present data can be compared with results from [4] and [3]. Both research groups subjected a PCA-determined number of sources to an unrestricted moving dipole fit procedure using a BESA algorithm. Both groups identified a pair of bilateral temporal and also frontal sources. Up to this point, our analysis shows results which are consistent with the finding of

both research groups, although we used a fairly different source modeling method (Figure S1A). This consistence only pertains to the topography. It should be noted that there are morphological differences. Specifically, we observed a bimodal course of activity in the frontal as well as in the temporal sources. In the prior report of [3], this bimodality was only present in the temporal source. There is also a deviance in the morphology between the sensor signals and the estimated source waveforms within our own data. Specifically, the peak activity arose slightly earlier in the estimated sources than in the raw sensor data (cf. Figures 1B and S1B). Apart from these reservations, the convergence of the comparisons drawn above permits a sufficient confidence into the calculation to interpret the results as reflecting a Nd. Given our expectations, acute stress would reduce source activity with a preponderance of this reduction in frontal sources (See Introduction). This expectation is met. Surprisingly, there was no confinement of this effect to a later processing stage. To summarize, the results of the source space analysis are in accordance with existing studies as well as with the aforementioned analysis based on raw sensor data, except for several deviations pertaining to the time course. Note, however, that the source space reconstruction was not the primary goal of the present study. The technical setup of the recordings was not optimized for this purpose due to a sparse sensor density and a limited cap coverage of the head. Source modeling is not only sensitive to computation modes, but also to the recording facilities used (c.f. [5–7]), such that conclusions should not solely be drawn on these results.

References

1. Grech R, Cassar T, Muscat J, Camilleri KP, Fabri SG et al. (2008) Review on solving the inverse problem in EEG source analysis. J Neuroeng Rehabil 5.
2. Peyk P, Schupp HT, Elbert T, Junghoefer M (2008) Emotion processing in the visual brain: A MEG analysis. Brain Topogr 20 (4): 205–215.
3. Jemel B, Oades RD, Oknina L, Achenbach C, Röpcke B (2003) Frontal and temporal lobe sources for a marker of controlled auditory attention: the negative difference (Nd) event-related potential. Brain Topogr 15 (4): 249–262.
4. Dien J, Tucker DM, Potts G, Hartry-Speiser A (1997) Localization of auditory evoked potentials related to selective intermodal attention. J Cognitive Neurosci 9 (6): 799–823.
5. Junghöfer M, Peyk P, Flaisch T, Schupp HT (2006) Neuroimaging methods in affective neuroscience: Selected methodological issues. Prog Brain Res (156): 123–143.
6. Junghofer M, Elbert T, Tucker DM, Braun C (1999) The polar average reference effect: a bias in estimating the head surface integral in EEG recording. Clin Neurophysiol 110 (6): 1149–1155.
7. Junghofer M, Elbert T, Leiderer P, Berg P, Rockstroh B (1997) Mapping EEG-potentials on the surface of the brain: A strategy for uncovering cortical sources. Brain Topogr 9 (3): 203–217.
